# Deciphering the impact of senescence in kidney transplant rejection: An integrative machine learning and multi-omics analysis via bulk and single-cell RNA sequencing

**DOI:** 10.1371/journal.pone.0312272

**Published:** 2024-11-27

**Authors:** Xihao Shen, Jiyue Wu, Feilong Zhang, Qing Bi, Zejia Sun, Wei Wang

**Affiliations:** 1 Department of Urology, Beijing Chao-Yang Hospital, Capital Medical University, Beijing, China; 2 Institute of Urology, Capital Medical University, Beijing, China; Rutgers: Rutgers The State University of New Jersey, UNITED STATES OF AMERICA

## Abstract

**Background:**

The demographic shift towards an older population presents significant challenges for kidney transplantation (KTx), particularly due to the vulnerability of aged donor kidneys to ischemic damage, delayed graft function, and reduced graft survival. KTx rejection poses a significant threat to allograft function and longevity of the kidney graft. The relationship between senescence and rejection remains elusive and controversial.

**Methods:**

Gene Expression Omnibus **(**GEO) provided microarray and single-cell RNA sequencing datasets. After integrating Senescence-Related Genes (SRGs) from multiple established databases, differential expression analysis, weighted gene co-expression network analysis (WGCNA), and machine learning algorithms were applied to identify predictive SRGs (pSRGs). A cluster analysis of rejection samples was conducted using the consensus clustering algorithm. Subsequently, we utilized multiple machine learning methods (RF, SVM, XGB, GLM and LASSO) based on pSRGs to develop the optimal Acute Rejection (AR) diagnostic model and long-term graft survival predictive signatures. Finally, we validated the role of pSRGs and senescence in kidney rejection through the single-cell landscape.

**Results:**

Thirteen pSRGs were identified, correlating with rejection. Two rejection clusters were divided (Cluster C1 and C2). GSVA analysis of two clusters underscored a positive correlation between senescence, KTx rejection occurrence and worse graft survival. A non-invasive diagnostic model (AUC = 0.975) and a prognostic model (1- Year AUC = 0.881; 2- Year AUC = 0.880; 3- Year AUC = 0.883) for graft survival were developed, demonstrating significant predictive capabilities to early detect acute rejection and long-term graft outcomes. Single-cell sequencing analysis provided a detailed cellular-level landscape of rejection, supporting the conclusions drawn from above.

**Conclusion:**

Our comprehensive analysis underscores the pivotal role of senescence in KTx rejection, highlighting the potential of SRGs as biomarkers for diagnosing rejection and predicting graft survival, which may enhance personalized treatment strategies and improve transplant outcomes.

## Introduction

Globally, the demographic shift towards an increased population over 65 has profound implications for kidney transplantation [1]. Aged donor kidneys are notably susceptible to ischemic injury, exhibit delayed functional recovery, and present reduced graft longevity [2–4]. This vulnerability is predominantly attributed to cellular senescence, hallmarked by a pro-inflammatory and tissue-modifying secretory profile, the Senescence-Associated Secretory Phenotype (SASP) [5]. This phenotype compromises renal tissue integrity and hampers regenerative processes, intensifying ischemia-reperfusion injury during transplantation [6]. Strategically targeting cellular senescence with anti-senescence pharmacological agents holds the promise of augmenting donor kidney quality and subsequently, the success rates of long-term transplant outcomes [7].

Notwithstanding the strides in immunosuppressive therapy, allograft rejection remains a pivotal impediment to graft longevity, precipitating functional deterioration and graft failure [8]. The intricate pathogenesis of kidney transplant rejection necessitates further elucidation. Currently reliant on histological biopsy findings, there is an imperative for non-invasive, precise biomarkers for rejection diagnostics. The role of senescence in this context remains unclear. Published reports indicate a dichotomy: Data derived from a multivariate analysis involving over 100,000 kidney transplant patients suggests a diminished rejection risk in older recipients, while other findings point towards an elevated risk within this demographic [9–12]. Moreover, research probing the nexus between aging and rejection predominantly gravitates towards clinical investigations, with a notable dearth of studies specifically exploring cellular senescence.

The use of computational models in transplant medicine has expanded significantly in recent years, with an increasing number of studies harnessing machine learning (ML) and bioinformatics tools to predict graft rejection and survival outcomes. For example, Wu et al. applied ML to analyze neutrophil extracellular trap-related genes (NRGs) in ischemia-reperfusion injury (IRI), identifying subtypes and developing predictive strategies for delayed graft function (DGF) and graft survival [13]. Similarly, Bi et al. utilized ML to pinpoint necroinflammation-associated biomarkers, resulting in highly accurate predictive tools for DGF and graft failure [14]. These studies highlight the potential of computational approaches to enhance transplant prognosis and personalize treatment strategies. However, research has yet to explore the use of cellular senescence as a predictive factor for graft rejection and survival through ML methods.

In this study, we totally integrated 1,186 senescence-related genes (SRGs). From a discovery cohort of 282 samples (GSE21374), we identified 33 senescence-related hub genes using differential gene expression and WGCNA analysis. Machine learning methods Supported Vector Machine Recursive Feature Elimination (SVM-RFE) and Random Forest (RF) were employed to refine this to 13 pSRGs, which classified samples into two clusters through consensus clustering. The expression of these pSRGs was validated in peripheral blood samples with acute graft rejection (GSE15296, GSE14067), leading to the identification of four non-invasive diagnostic signatures. These were utilized to construct robust diagnostic models and a prediction nomogram. To explore the relationship between senescence and graft prognosis, we employed a Unicox-Lasso approach, identifying four prognostic SRGs and developing a strong prognostic model. Additionally, the dataset (GSE189536) facilitated the construction of a single-cell landscape for kidney transplant rejection, examining cellular-level expression of prognostic SRGs and further affirming the role of senescence in rejection. Overall, our comprehensive bioinformatics analysis highlighted the significant role of cellular senescence in kidney transplant rejection and elucidated its diagnostic and prognostic implications.

## Materials and methods

### Dataset acquisition and processing

This study compiled SRGs from the CellAge, Aging Atlas, and GenAge databases [15–17]. Table 1 summarizes detailed information of the 6 datasets used in our study. In short, the discovery cohort, GSE21374 [18], included RNA-seq data from 76 rejection and 206 non-rejection biopsy samples. Validation was performed using datasets GSE129166 [19] and GSE192444 [20]. Peripheral blood microarray profiles (GSE15296 [21], GSE14067 [22]) aided in developing rejection diagnostic models. Single-cell data from GSE189536 [23].

**Table 1:** 6 data sets included in this study.

Data set	Tissue	Platforms	Sample	Application
GSE21374	KTx biopsy	Affymetrix Human Genome U133 Plus 2.0 Array	76 Rejection 206 Non-rejection	Bulk data set analysis Graft survival analysis
GSE129166	KTx biopsy	Affymetrix Human Genome U133 Plus 2.0 Array	35 ABMR/TCMR 60 Non-rejection	Validation data set
GSE192444	KTx biopsy	Affymetrix Human Genome U219 Array	125 Rejection 175 Non-rejection	Validation data set
GSE15296	Peripheral blood	Affymetrix Human Genome U133 Plus 2.0 Array	51 AR 24 STA	AR diagnostic analysis
GSE14067	Peripheral blood	Affymetrix Human Genome U133 Plus 2.0 Array	60 AR 62 STA	Diagnostic model validation
GSE189536	KTx biopsy	Illumina NovaSeq 6000	9 Rejection 2 Non-rejection	scRNA analysis

### Identification of senescence-related hub genes

DEGs in the GSE21374 dataset were pinpointed using R package "Limma" (version 3.56.2), with significant changes identified by an adjusted p-value below 0.05 and an absolute log fold change greater than 0.5 [24]. The adjusted p-values were calculated using the Benjamini-Hochberg (BH) method to control the false discovery rate (FDR). WGCNA analysis via the R package "WGCNA" determined the module most associated with allograft rejection [25]. Intersection of DEGs, optimal module genes, and SRGs yielded hub DE-SRGs, delineating differences between rejection and control samples.

### Machine learning methods screening predictive SRGs

We applied two machine learning techniques, the RF and SVM-RFE, to identify SRGs predictive of rejection. RF, a supervised ensemble of decision trees, was utilized via the "randomForest" R package, ranking features by Gini importance [26]. SVM-RFE, executed through the "e1071" R package, employs a recursive feature elimination strategy, optimizing classification accuracy [27]. In RF, the top features were selected based on their Gini importance, with higher-scoring genes considered more critical for classification. In SVM-RFE, features were selected by minimizing classification error, ensuring that only the most predictive genes were retained. To ensure robustness, we selected overlapping features from both models, reducing the potential for model-specific biases. This intersection approach was chosen to enhance the reliability of the identified SRGs, as only features consistently important across both methods were considered. The intersection of top-ranked RF genes and SVM-RFE genes resulted in the identification of 13 critical SRGs.

### Consensus clustering

To determine distinct senescence subtypes, the "ConsensusClusterPlus" R package was used to analyze the GSE21374 dataset focusing on the 13 pSRG expression [28]. Cluster numbers (k) ranged from 2 to 5. The "partitioning around medoids" algorithm, paired with the "1-Spearman correlation" distance measure, was used for cluster analysis. This method involved resampling 80% of the dataset for 50 iterations, ensuring robustness and reliability in identifying distinct clusters based on sample similarities. Optimal cluster number selection (k) was based on achieving the highest consensus within each cluster.

### Establishment of the non-invasive diagnostic model

Four pSRGs differently expressed in two AR peripheral blood samples were utilized to construct models using RF, General Linear Model (GLM), Support Vector Machine (SVM), and Extreme Gradient Boosting (XGBoost) via the following R packages: “caret”, “DALEX”, “kernlab”, “xgboost”, “randomForest” [26,29–32]. The optimal model was selected using ROC curves and AUC values. Additionally, a nomogram was developed for rejection progression prediction, with its accuracy assessed through a calibration curve and its clinical applicability evaluated via the ROC curve.

### Construction of the graft survival predictive model

To assess senescence’s effect on allograft longevity, Univariable Cox regression initially filtered SRGs for long-term prognosis, using the “survival” R package [33]. GSE21374 samples were then split (7:3) into training and testing sets for least absolute shrinkage and selection operator (LASSO) regression analysis to derive a predictive signature [34]. This involved calculating risk scores (Riskscore = *∑n i = 1[geneCoef*_*i*_ *× geneExp*_*i*_*]*) to distinguish between high-risk and low-risk groups, with their prognostic differences evaluated through time-dependent ROC curves, Kaplan-Meier analysis, risk curves, and Chi-square tests.

### scRNA-seq data analysis

R Package "Seurat" was employed for the analysis of single-cell genomics data [35]. We systematically excluded cells exhibiting less than 200 or more than 5000 genes, as well as those whose mitochondrial gene content exceeded 25%. Through the "FindVariableFeatures" function, genes exhibiting high variability were identified, followed by principal component analysis (PCA). A uniform manifold approximation and projection (UMAP) method was used to reduce dimensionality. The "FindAllMarkers" function was utilized to conduct differential expression analysis, applying a threshold of an absolute log2 fold-change greater than 0.25 and an adjusted p-value below 0.05. Annotations for single-cell subgroups were derived from published literatures and "CellMarker2.0" database [23,36–38]. The "FeaturePlot" function was employed for gene expression visualization and the "plot1cell" R package facilitated the depiction of single-cell plots [39]. "CellChat" package was utilized to analyze cellular interactions, while the "AUCell" package computed the senescence score of each cell based upon the gene set "REACTOME_CELLULAR_SENSCENCE" from the Molecular Signatures Database (MSigDB; http://www.gsea-msigdb.org/gsea/msigdb/index.jsp) [40,41].

### Analysis of functional enrichment and immune characteristics

To discern differences between normal and KTx rejection samples in senescence-related biological processes, Gene Set Enrichment Analysis (GSEA) was conducted. R package “Clusterprofiler” was employed to analyze the biological functions and pathways of senescence-related hub genes using Gene Ontology (GO) and Kyoto Encyclopedia of Genes and Genomes (KEGG) pathway enrichment analyses [42]. Using R package "GSVA" for gene set variation analysis, we referenced hallmark gene sets (h.all.v2023.1.Hs.symbols.gmt) and manually curated aging/senescence-related gene sets (S1 Table) from MSigDB to pinpoint biological pathway variances across rejection subtypes [43]. Functions and pathways with a FDR below 0.05 were deemed significantly enriched. Single sample gene set enrichment analysis (ssGSEA) was used to quantify immune response activities and populations through "GSVA" and "GSEABase" R packages, analyzing immune abundance and activity in single samples [44].

### Statistical analysis

The statistical analyses conducted in this study were carried out using R software (version 4.3.1). A descriptive statistic gives an overview of distributions for continuous variables, including averages, medians, quartiles, ranges, standard deviations, and standard errors, as well as for nominal variables, which were summarized by frequency and percentage. For normally distributed variables, the Student’s t-test assessed group differences. The Mann-Whitney U test analyzed non-normally distributed variables. Associations between rejection types and risk categories were examined with the Chi-square test, enabling statistical analysis across various data distributions and categorical relationships. Statistical significance was determined by a p-value less than 0.05 for all two-sided tests.

## Results

### Hub SRGs identification and functional enrichment analysis

**Fig 1:**
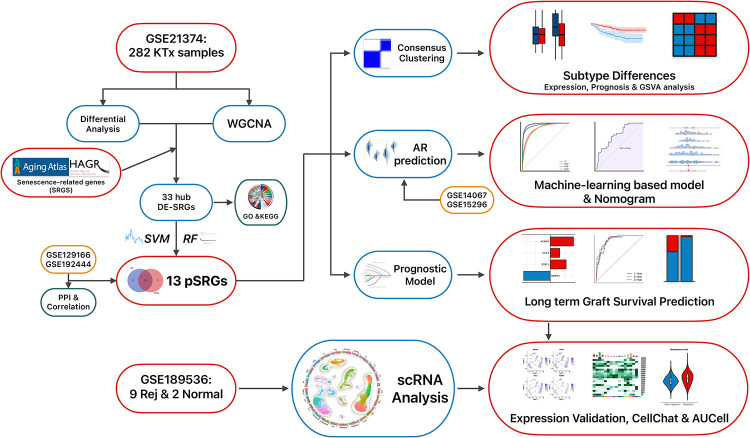
The flowchart illustrates the methodology employed in this study.

**Fig 2:**
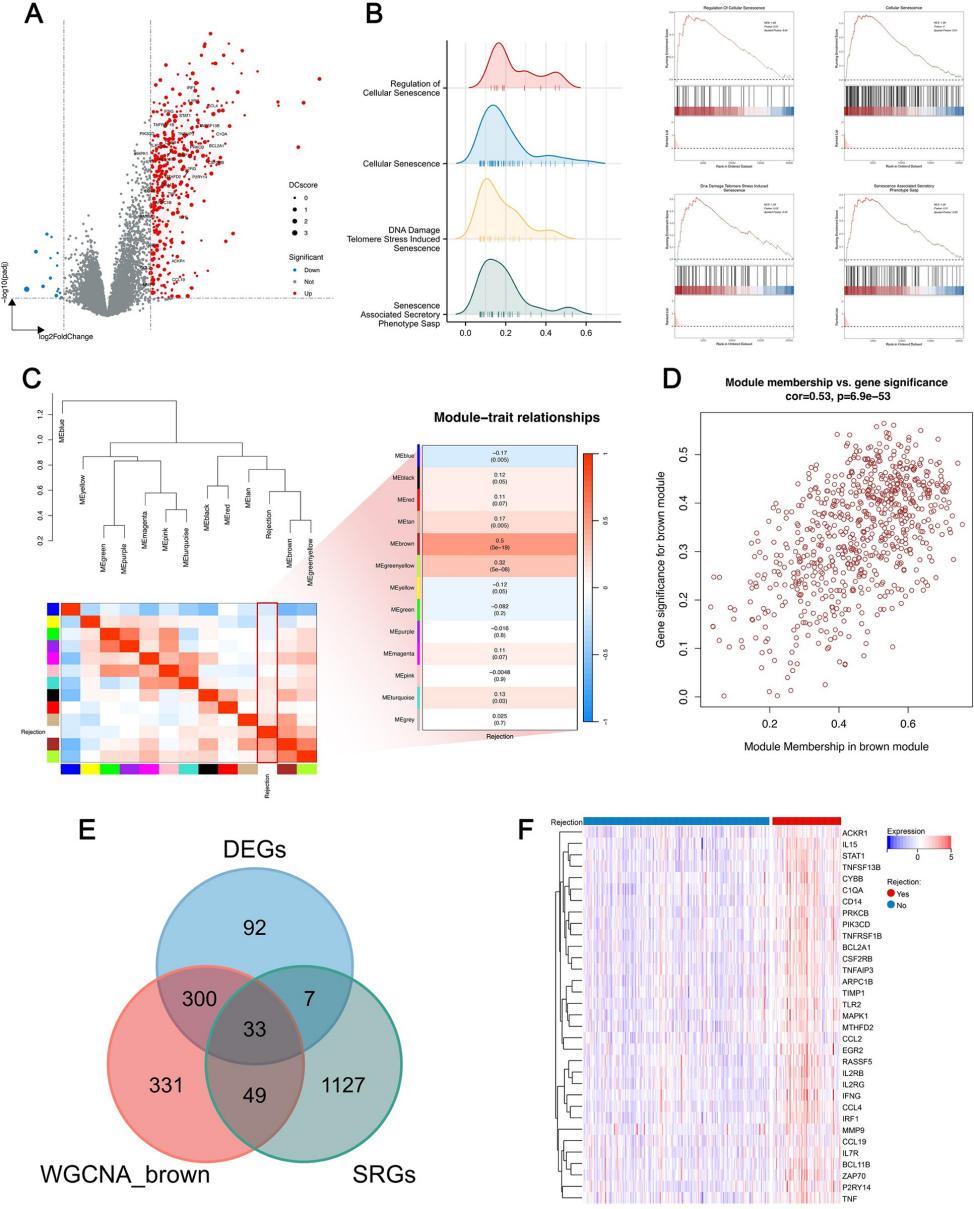
Identification of hub DE-SRGs. **A.** Volcano plot of DEGs, with gene symbols of hub DE-SRGs labeled and red lines illustrating the PPI between them. The size of each point indicates the gene’s importance within the PPI network. **B.** GSEA analysis demonstrating senescence-related pathways differently enriched between normal and rejection samples. **C.** Correlation heatmaps between different modules and rejection via WGCNA analysis. **D.** Correlation analysis of the module brown displaying the module connectivity of genes on the x-axis against the correlation coefficient with the phenotype on the y-axis. **E.** Intersection of DEGs, SRGs and genes from the brown module yielding 33 hub DE-SRGs. **F.** Heatmap of 33 hub DE-SRGs expression profiles in normal and rejection samples.

The workflow of this study is depicted in Fig 1. To investigate the effect of cellular senescence on KTx rejection, gene expression profiles from 206 control samples and 76 rejection samples were analyzed for differential expression. Following the selection criteria mentioned, 416 upregulated and 17 downregulated genes were obtained (Fig 2A). Subsequently, GSEA analysis utilized the GO and REACTOME gene sets from MSigDB. The results revealed an enrichment of the rejection samples in pathways related to cellular senescence (Fig 2B). Moreover, WGCNA analysis was utilized to identify gene modules most correlated with rejection (Figs 2C and S1). A heatmap indicated the highest correlation (0.5) with rejection in the brown module, designating it as the key module (Fig 2C). The scatterplot revealed a strong correlation between gene significance and module membership within the module (Fig 2D). Intersection of the DEGs, genes from the brown module, and SRGs yielded 33 hub DE-SRGs (Fig 2E), whose expression heatmap is displayed in Fig 2F. These 33 genes are also marked on the volcano plot, where red lines represent their complex protein-protein interactions (PPI), and the size of each point reflects the Degree Centrality (DCscore), measuring the importance of each protein in the PPI network (Fig 2A).

**Fig 3:**
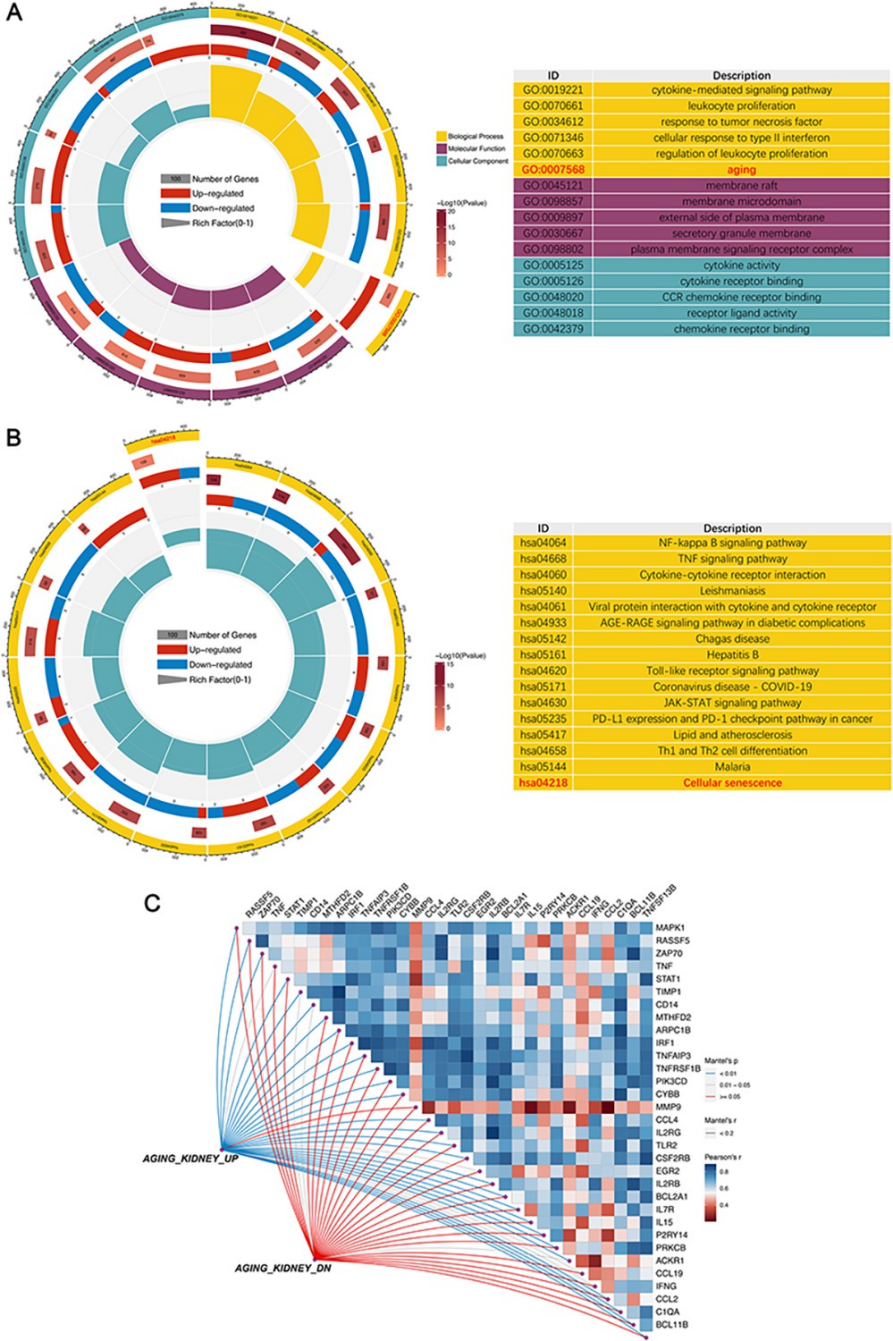
Functional enrichment and correlation analysis of hub DE-SRGs. **A.** GO enrichment analysis of hub DE-SRGs. BP: Biological process; CC: Cellular component, MF: Molecular function. **B.** KEGG pathway analysis of hub DE-SRGs. **C.** The analysis of the correlation among the 33 genes and their correlation with “AGING_KIDNEY” datasets.

To shed light on the potential role of hub DE-SRGs in rejection, we performed GO and KEGG analyses on these 33 genes, revealing significant enriched pathways, as shown in Fig 3A and 3B. Notably, significant enrichment was observed across aging/senescence-related pathways. Furthermore, gene sets "AGING_KIDNEY_UP" and "AGING_KIDNEY_DOWN," comprising differentially expressed genes associated with aging kidneys, were retrieved from the MSigDB. A Mantel test displaying the correlation analysis between the 33 hub DE-SRGs and these public gene sets revealed a significant positive correlation with the "UP" set for all but MMP9, validating the strong association of the 33 hub genes with aging (Fig 3C).

### Screening of rejection predictive SRGs

**Fig 4:**
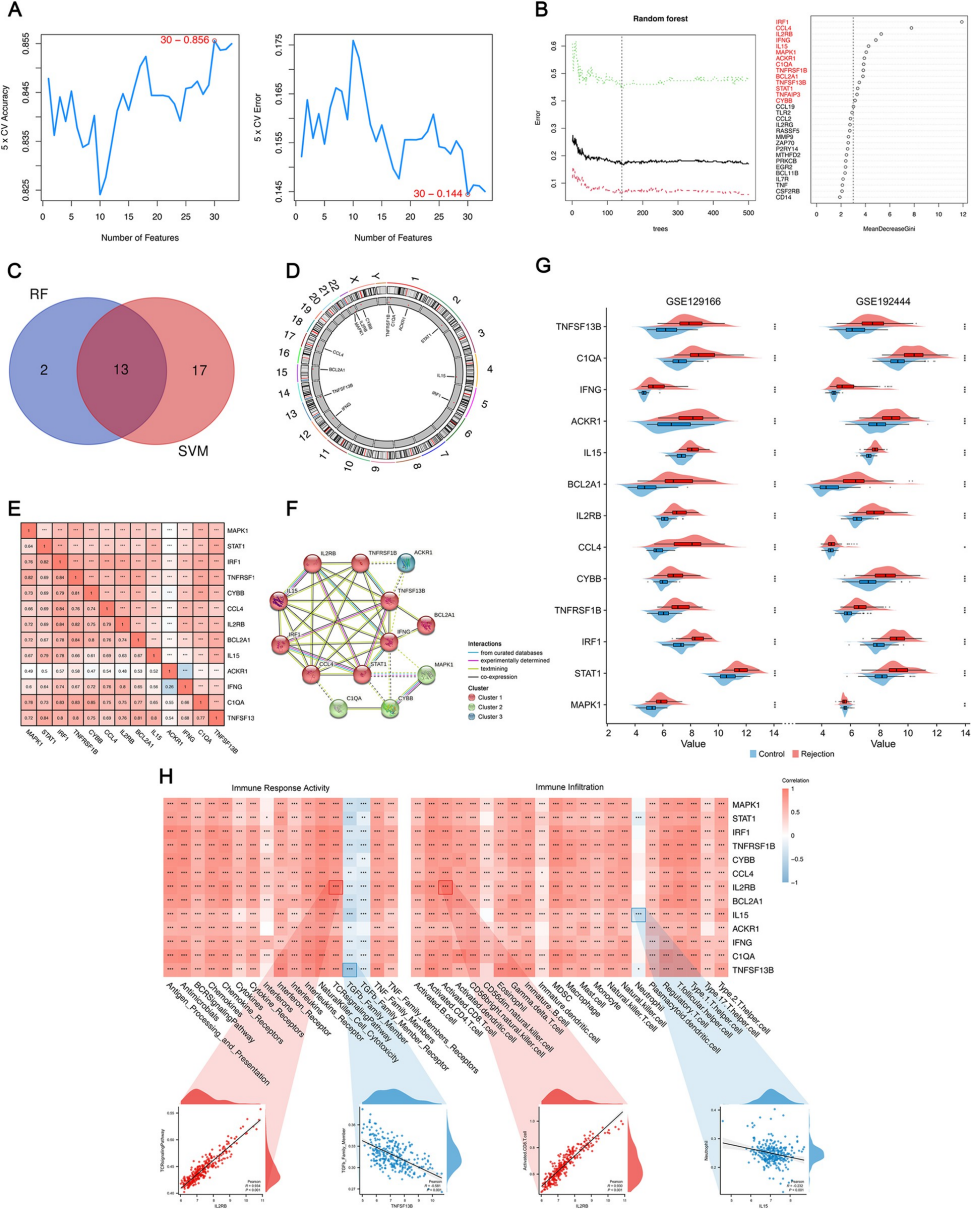
Features of 13 pSRGs obtained by machine learning methods. **A.** SVM-RFE algorithm selecting 30 SRGs at the optimal point. **B.** Random forest tree plot and corresponding Gini importance measure. The tree holding the minimum error rate was marked by a dotted line. **C.** 13 pSRGs obtained by intersecting sets of genes by applying two machine learning algorithms. **D.** The chromosomal positions of the 13 pSRGs. **E.** Correlation analysis of 13 pSRGs. **F.** Protein–protein interactions among pSRGs. **G.** Validation of the differential expression of the 13 pSRGs using GEO129166 and GSE192444. **H.** Correlation between the pSRGs and immune characteristics.

The escalating scale and inherent complexity of biological data have spurred the increasing use of machine learning in medicine, aimed at constructing models that are both informative and predictive of the fundamental biological processes [45]. Here, we employed SVM-RFE and RF methodologies to identify predictive signatures for KTx rejection. As depicted in Fig 4A, the SVM algorithm, validated through 5-fold cross-validation, achieved its highest accuracy (0.856) and lowest error rate (0.144) with a set of 30 genes. Meanwhile, Fig 4B illustrates the outcome of the RF method, where we selected 15 genes with Gini values exceeding 3. An intersection of genes identified by both machine learning algorithms yielded 13 final pSRGs (Fig 4C and Table 2). The chromosomal positions of these genes are shown in Fig 4D. Fig 4E reveals their interrelationships, highlighting the weakest correlation between ACKR1 and IFNG, and the strongest between C1QA and CYBB. Additionally, we performed a Protein-Protein Interaction (PPI) analysis using Cytoscape (https://cytoscape.org) (Fig 4F). To validate the reliability of these 13 pSRGs, we incorporated two external datasets, confirming differential expression in all genes (Fig 4G). Investigating transplant immunology provides profound insights into the mechanisms underlying rejection events. Herein, ssGSEA analysis was utilized to potentially uncover the immunological factors influenced by pSRGs in rejection. In the analysis of correlation with immune response activities, IL2RB exhibited the strongest positive correlation with TCR, while TNFSF13B showed the most negative correlation with TGF-β. Immune infiltration analysis revealed that IL15 had the most negative correlation with Neutrophils, and IL2RB had the strongest positive correlation with activated CD8^+^T cells (Fig 4H).

**Table 2:** 13 rejection predictive senescence-related genes(pSRGs).

Symbol	Name
CYBB	cytochrome b-245 beta chain
ACKR1	atypical chemokine receptor 1 (Duffy blood group)
IFNG	interferon gamma
IL2RB	interleukin 2 receptor subunit beta
C1QA	complement C1q A chain
TNFSF13B	TNF superfamily member 13b
BCL2A1	BCL2 related protein A1
IRF1	interferon regulatory factor 1
MAPK1	mitogen-activated protein kinase 1
TNFRSF1B	TNF receptor superfamily member 1B
CCL4	C-C motif chemokine ligand 4
STAT1	signal transducer and activator of transcription 1
IL15	interleukin 15

### Stratification of kidney rejection patients according to pSRGs

**Fig 5:**
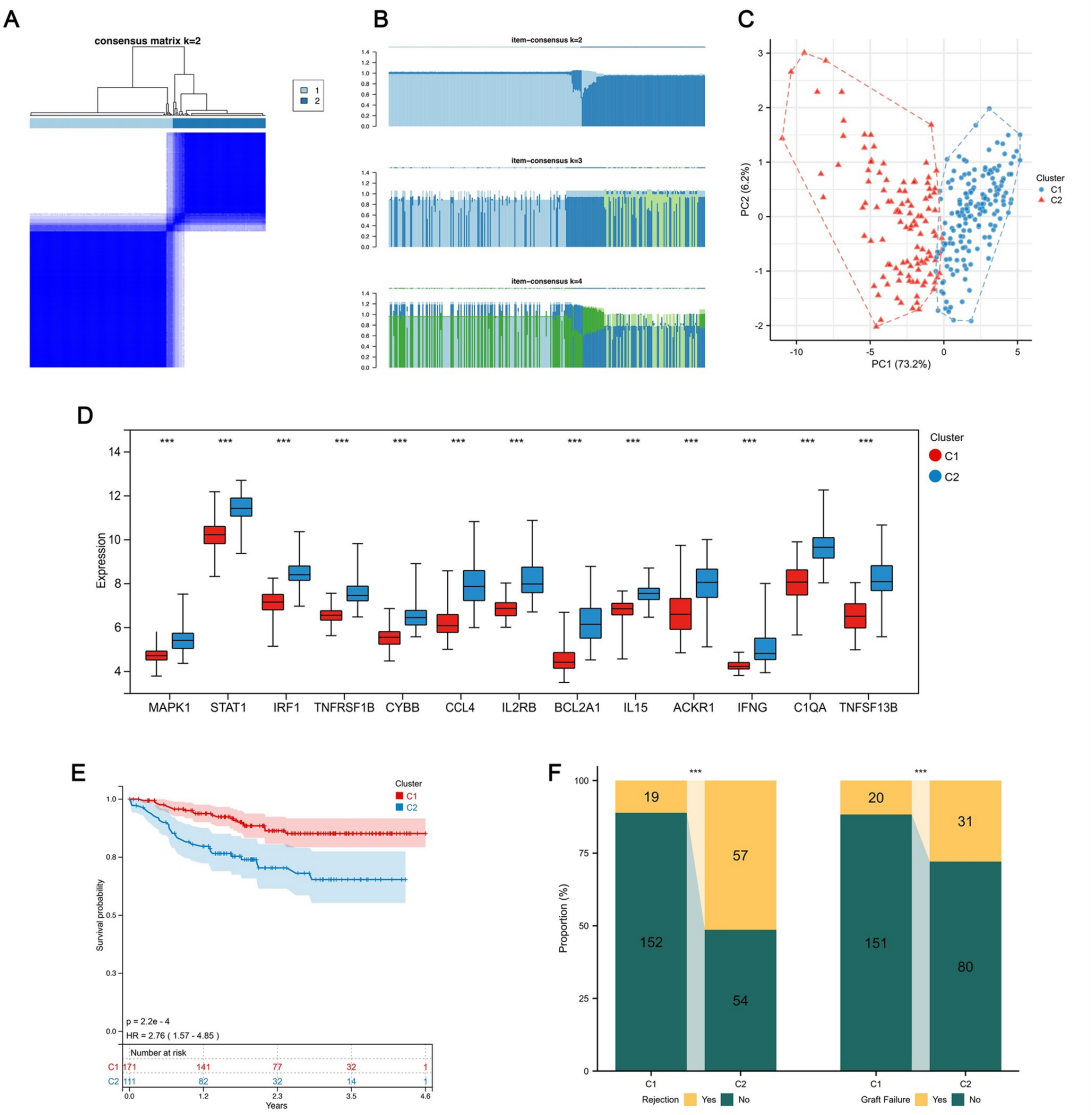
Discovering two KTx rejection subtypes. **A.** Consensus clustering map for k = 2. **B.** Consensus of the items in each cluster (k = 2–4). **C.** PCA plot depicting pSRG expression patterns across the two clusters. **D.** Box plot illustrating expression disparities of the 13 pSRGs between the two clusters. **E.** K-M analysis plot demonstrating enhanced overall survival of cluster C1 compared to C2. **F.** Stacked bar charts showcasing variations in rejection proportions and graft survival.

A consensus clustering analysis based on the expression profiles of 13 pSRGs was conducted to distinguish kidney transplant recipients with varying levels of rejection. We focused on achieving unanimity within the clusters to ascertain the ideal cluster count (k), guided by the consensus clustering outcomes (Fig 5B). Ultimately, all samples were categorized into two specific subtypes (Fig 5A): Cluster C1, consisting of 171 samples, and Cluster C2 with 111 samples, with this division being more definitive than other evaluated values for k. In the two rejection subtypes, SRG expression diverged significantly by PCA analysis (Fig 5C), with gene expression disparities illustrated through a box plot (Fig 5D). Notably, all 13 pSRGs were found to be upregulated in C2, indicating a greater degree of cellular senescence. Kaplan-Meier analysis demonstrated that patients in cluster C2 had a poorer overall prognosis (Fig 5E). Similarly, in terms of clinical characteristics, individuals in C2 experienced a higher rate of rejection and a lower success rate of transplantation (Fig 5F).

### GSVA enrichment and immunity analysis of senescence subtypes

**Fig 6:**
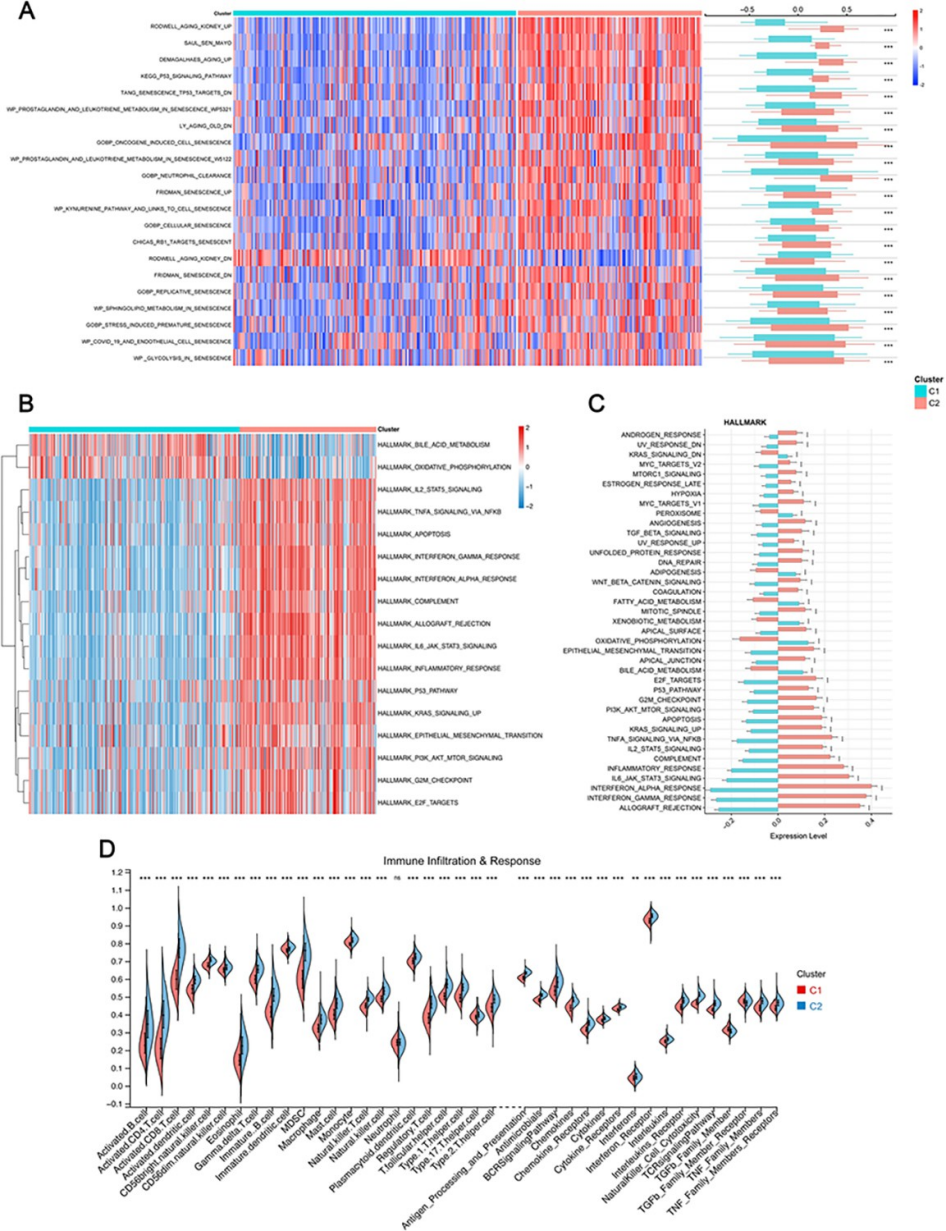
GSVA analysis and Immunological analysis. **A.** Differences of senescence/aging-related pathways enriched between two clusters. **B, C.** Heatmap (**B**) and bar plot (**C**) displayed distinct hallmark pathways between the two clusters. **D.** Subtype immune infiltration and response activity disparities.

To shed light on distinct senescence-related features between the two rejection clusters, GSVA enrichment analysis was performed utilizing senescence or aging-related datasets (S1 Table) manually compiled from the MSigDB Database. Out of 39 gene sets, 37 exhibited significant expression differences between clusters C1 and C2, with a pronounced enrichment in C2, as depicted by the heatmap and corresponding box plots (Fig 6A). Additionally, the hallmark gene set (h.all.v2023.1.Hs.symbols.gmt) was also used as the basis for GSVA analysis. As illustrated in Fig 6B and 6C, Cluster C2 showed significant links to pathways involved in allograft rejection, inflammation, and cell death, indicating a higher predisposition to negative transplant outcomes. In contrast, Cluster C1 was predominantly active in metabolic pathways, suggesting different biological focuses and potential implications for transplant health and patient management strategies.

Moreover, the ssGSEA immune signature analysis unveiled a stark contrast in immune activities between the two clusters. Cluster C2 exhibited marked upregulation in immune infiltration and activity compared to Cluster C1, indicating a more pronounced immune response that could influence the outcomes of transplantation. (Fig 6D).

### Construction and validation of peripheral blood AR predictive model

**Fig 7:**
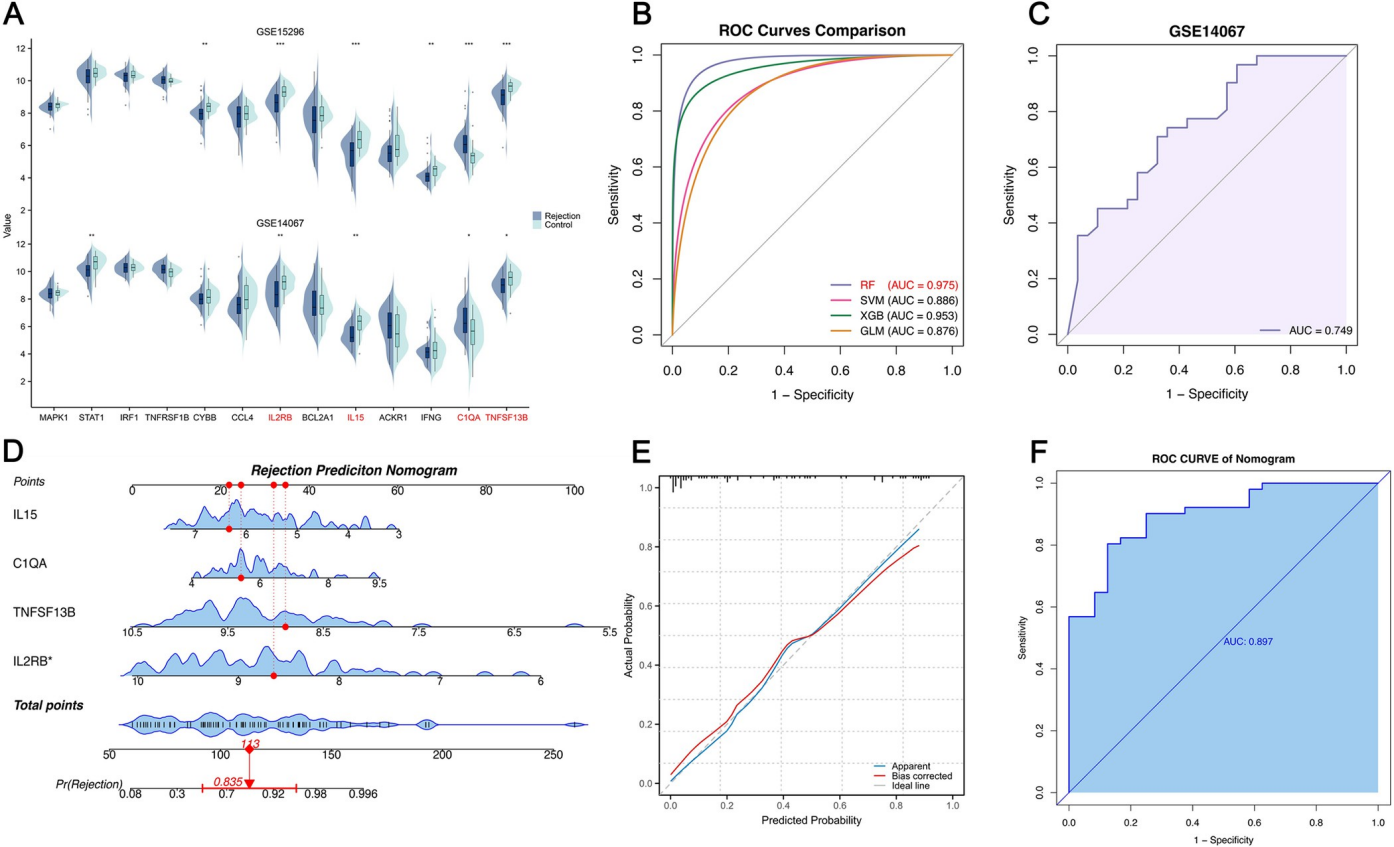
Construction of non-invasive diagnostic model and nomogram of AR. **A.** Expression profiles of 13pSRGs in peripheral blood datasets. Four genes that were differentially expressed in both datasets are highlighted in red. **B.** ROC curves evaluating the performances of the models. RF model was ultimately selected. **C.** Validation of the diagnostic model in the GSE14067. **D.** Rejection prediction nomogram based on the four pSRGs. **E.** Calibration curve of the nomogram. **F.** ROC curve demonstrating the effectiveness of the nomogram.

Acute Rejection significantly compromises graft survival. However, diagnosis relies on biopsies, making the construction of a non-invasive, efficient diagnostic signature crucial. Based on the expression of pSRGs in two peripheral blood datasets for AR, we selected 4 differentially expressed pSRGs (IL15, C1QA, TNFSF13B, and IL2RB) to construct machine learning-based diagnostic models (Fig 7A). Utilizing the dataset GSE15296, we employed RF trees, SVM, XGBoost, and GLM algorithms to develop diagnostic models. The RF model demonstrated superior performance, as evidenced by the smallest residuals in both the reverse cumulative distribution and box plots of the residuals, indicating its high accuracy and predictive reliability in the analysis. (S2 Fig). Additionally, the RF model presented AUC value of 0.975 (Fig 7B). Consequently, we selected the RF model and utilized the gene set GSE14067 to validate its diagnostic performance, achieving corresponding AUC values of 0.749 (Fig 7C). Furthermore, a nomogram incorporating the 4 pSRGs was developed to estimate the probability of AR (Fig 7D). Each variable is assigned a score, which are then added together to calculate a total score representing the sum of all individual scores. The error curve of the nomogram is depicted in Fig 7E, and ROC curve demonstrates its robust performance (Fig 7F).

### Investigating the role of SRGs in forecasting the longevity of transplanted kidneys

**Fig 8:**
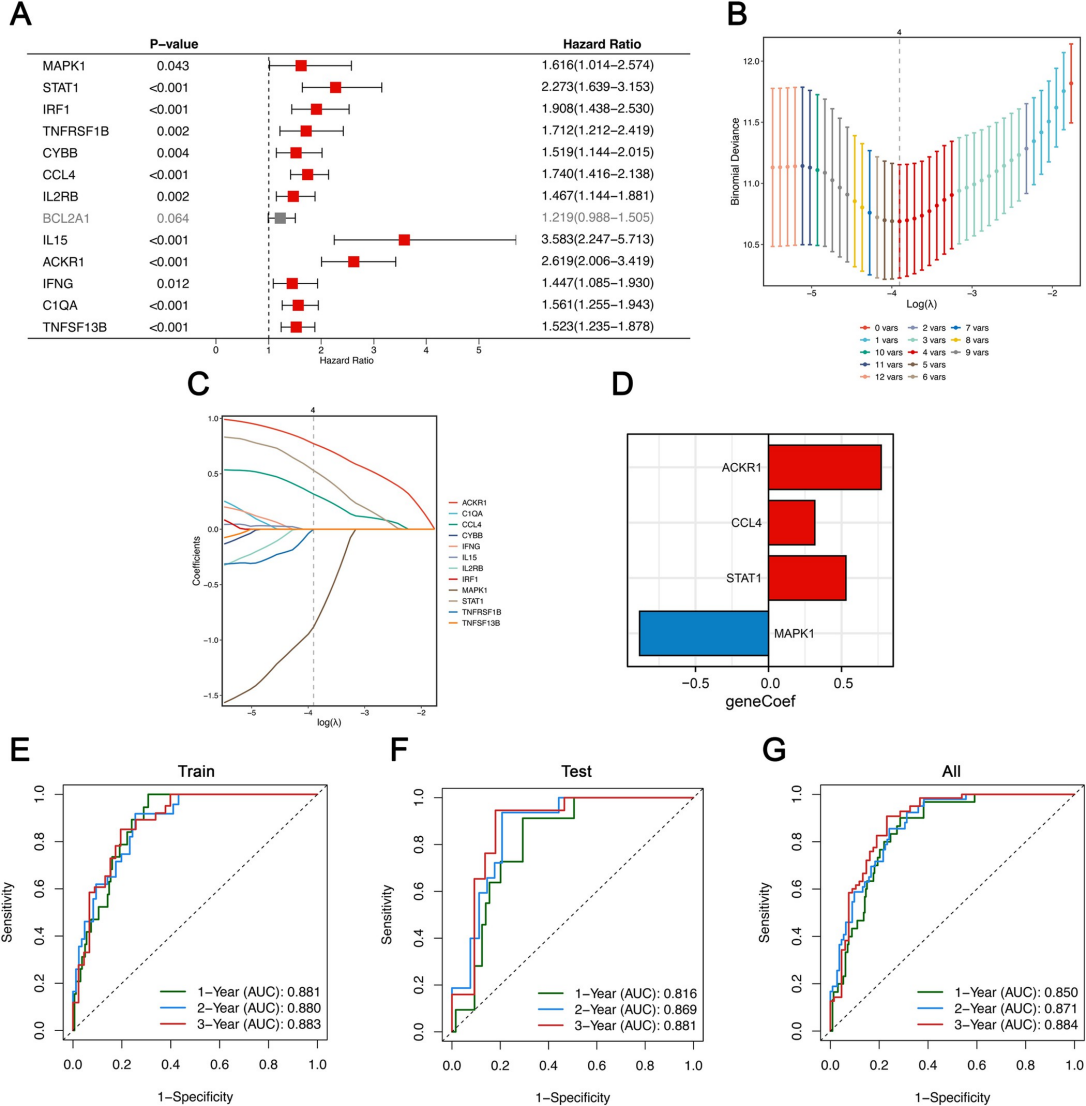
Establishment of a senescence-related graft survival prognostic model. **A.** Univariable Cox regression analysis screening prognostic SRGs. **B.** Ten-fold cross-validation selecting λ in LASSO model. **C.** LASSO coefficient profiles of 12 pSRGs. The optimal λ = 4 was selected. **D.** Coefficients of the candidate SRGs. **E-G.** Time-dependent ROC analysis in the training group (**E**), the testing group(**F**) and all(**G**).

To explore the predictive power of SRGs for graft survival after biopsy, we screened for long-term prognosis-related SRGs using Univariate Cox regression, the results of which are depicted in a forest plot (Fig 8A). Dividing recipients for training and testing (7:3 ratio), LASSO regression identified 4 key pSRGs (ACKR1, CCL4, STAT1, MAPK1; Fig 8B and 8C). Risk scores, based on these genes’ expression levels and coefficients, were calculated, with time-dependent ROC curves validating their predictive accuracy for 1–3 years survival (Fig 8D). These detailed AUC values—0.881, 0.880, and 0.883 for the training set and 0.816, 0.869, and 0.881 for the test set—illustrate the model’s robustness and reliability in forecasting long-term graft survival, highlighting its potential for clinical application in monitoring transplant outcomes (Fig 8E and 8G).

**Fig 9:**
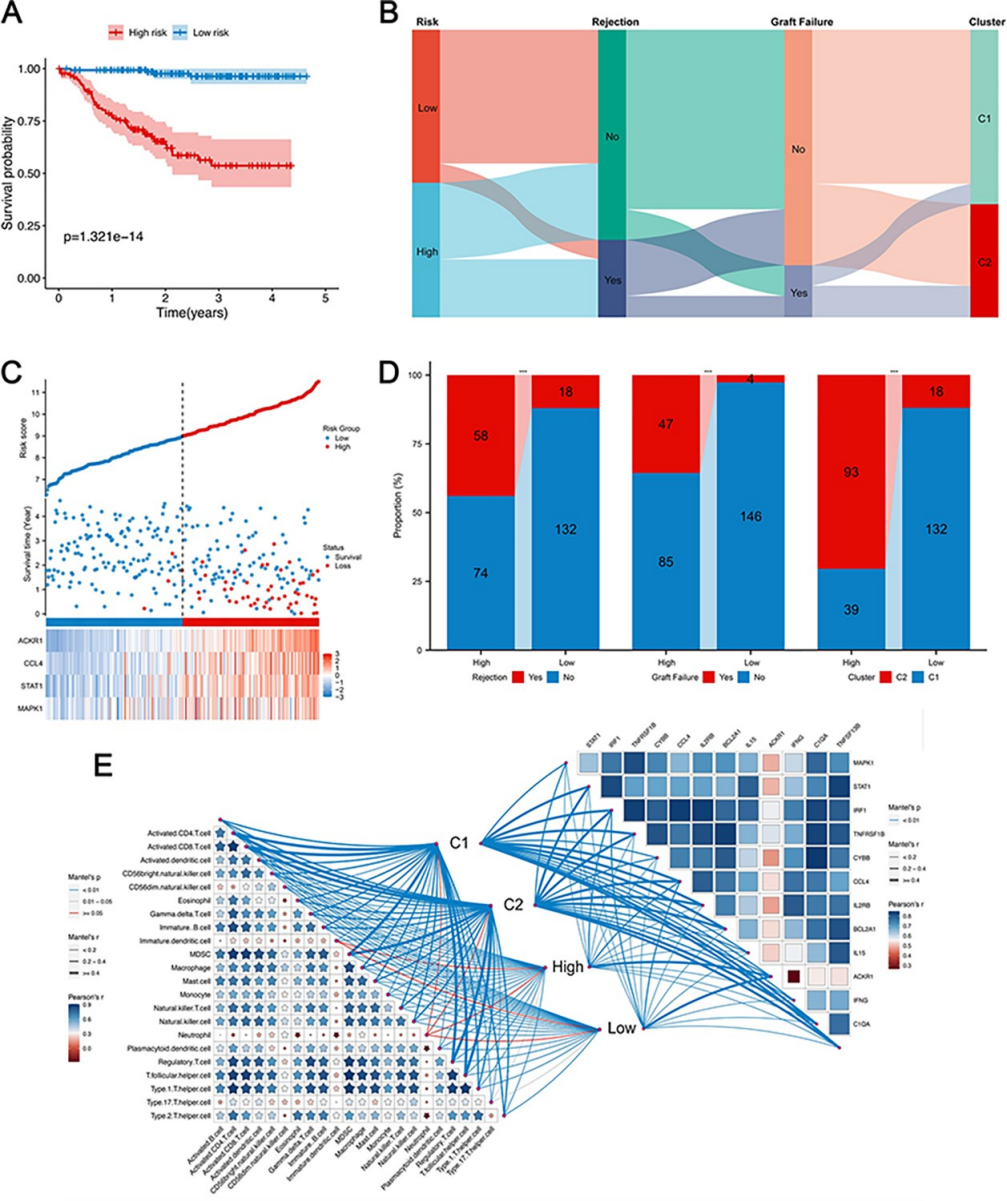
Clinical characteristic comparison. **A.** K–M plot of graft survival. **B.** Sankey diagram illustrating the correlation among recipient risk, rejection incidence, long-term graft failure, and clustering subtypes. **C.** Risk map of the different risk groups. **D.** Histogram illustrating the variances in rejection incidence, graft failure, and diverse clusters between the two groups. **E.** The interplay among pSRGs, immune-related cells, and their associations with rejection subgroups categorized by two distinct strategies.

Dividing patients by median risk scores revealed stark contrasts in graft rejection and loss between low- and high-risk groups, with high-risk patients showing worse outcomes (Fig 9C). The K–M curve clearly illustrated worsening outcomes in the high-risk group over time, suggesting that recipients with higher risk scores were more prone to experiencing graft rejection and loss (Fig 9A). Furthermore, a higher percentage of patients in the high-risk group were associated with Cluster C2. This division aligns with Cluster C2’s severe outcomes, underscoring the correlation between risk scores, immune cell dynamics, and rejection rates. (Fig 9D). The Sankey diagram summarized the different composition of the patient cohorts (Fig 9B). Fig 9E illustrates the intricate relationships among 22 immune-related cells, the interplay between pSRGs, and how both relate to different rejection subgroups.

### scRNA atlas of Senescence in kidney transplant rejection

**Fig 10:**
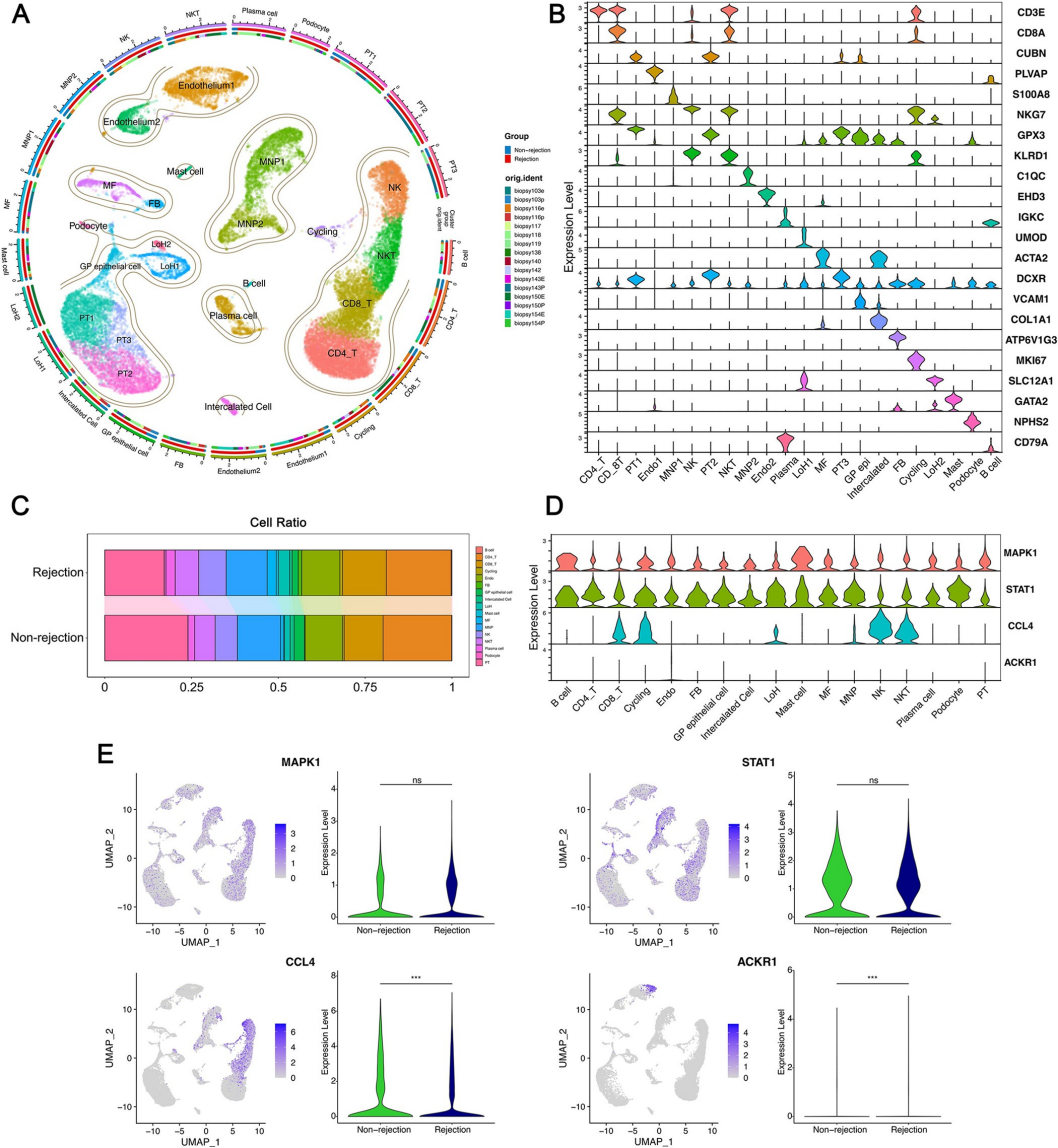
Single-cell analysis of KTx rejection and healthy individuals. **A.** Circular plot to visualize cell subpopulations, with different colors representing distinct subpopulations of cells. **B.** Violin plot utilized to display the marker gene expression. **C.** Bar plot illustrating cell subpopulation proportion in individuals with rejection and those without. **D.** Cellular expression level of four prognostic pSRGs. **E.** UMAP projection and violin plots illustrating the expression of pSRGs across different cell subpopulations and groups respectively.

**Fig 11:**
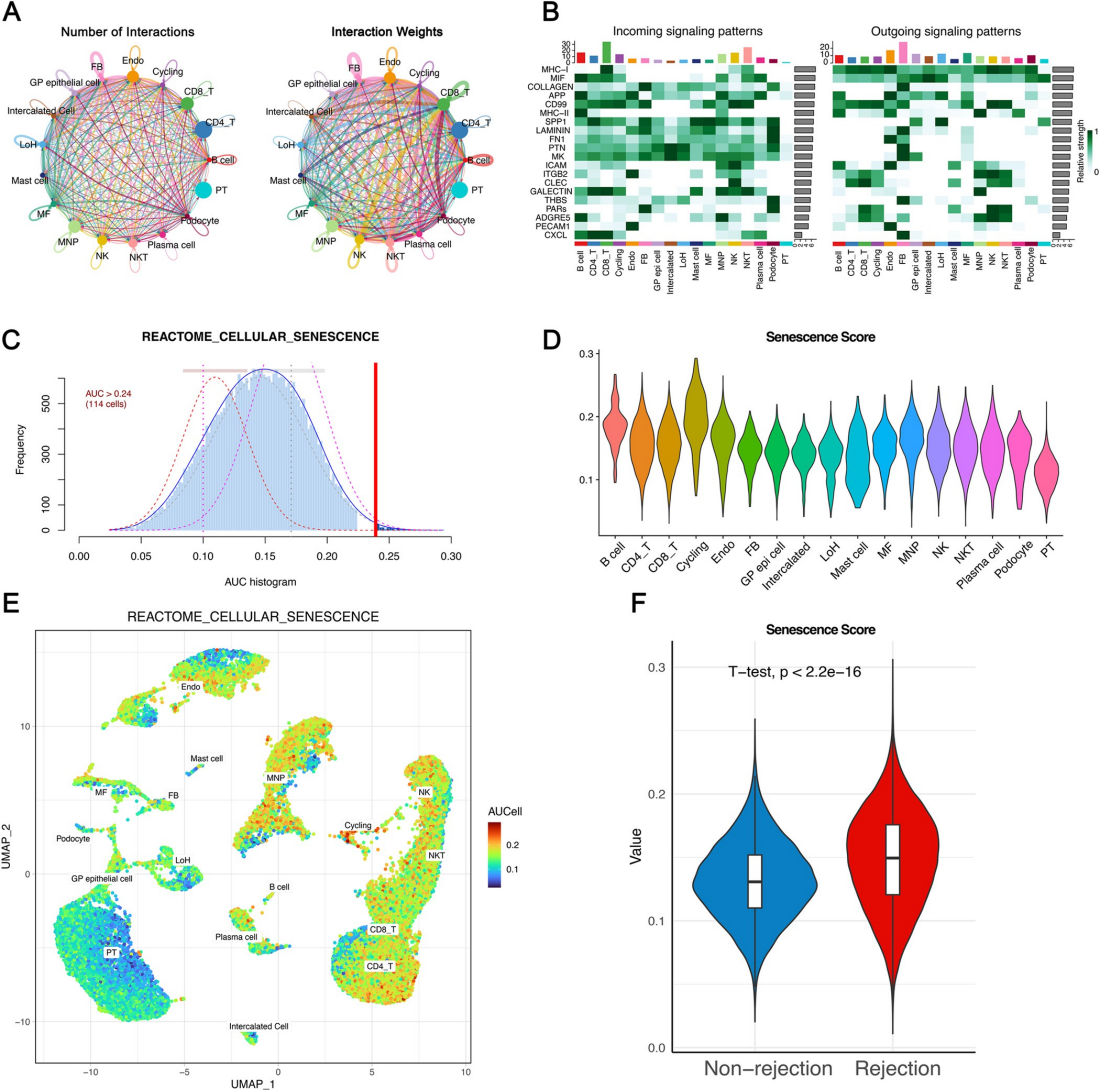
Cell communication and establishment of senescence score. **A.** The numbers and weights of cell communication by CellChat. **B.** Heatmap visualizing the input and output signaling pathways of various cell subpopulations. **C.** All cells were scored according to “REACTOME_CELLULAR_SENESCENCE” gene sets. **D-E.** Senescence score of different cell subpopulations visualized by (**D**) violin plot and (**E**) UMAP plot. **F.** Senescence scores significantly differed between the rejection and the non-rejection group.

Leveraging published single-cell transcriptome data (GSE189536), we further analyzed the significance of senescence. After meticulous quality control and filtering procedures, we obtained an expression matrix comprising 36,601 genes from 32,015 single cells (S3 Fig). By employing distinct marker genes for individual cell types, we classified the total cell population into 22 clusters representing 17 major cell types (Fig 10A and 10B). A box plot illustrated the differential proportions of rejection and non-rejection cell types (Fig 10C). We proceeded to validate the expression of four prognostic SRGs at the single-cell level; MAPK1 and STAT1 were expressed across multiple cell types, showing no differential expression between rejection and control groups. In contrast, CCL4 were predominantly expressed in CD8_T cells, Cycling cells, NK cells, and NKT cells and ACKR1 in Endothelium cells, with both exhibiting higher expression levels in the rejection group (Fig 10D and 10E). Subsequently, utilizing the CellChat package, we quantitatively explored the intricate intercellular interactions within the rejection cohort, extrapolating communications among various cell types (Fig 11A). The pivotal signals facilitating communication between different cell types were also delineated, with CD8_T cells showing the highest incoming signaling and fibroblasts (FB) the most outgoing (Fig 11B).

The "AUCell" R package was utilized to pinpoint cells actively participating in specific biological processes or pathways, facilitating the identification of cells involved in critical functions or disease states by analyzing their gene expression profiles. The “REACTOME_CELLULAR_SENESCENCE” dataset from MSigDB was inputted. Based on the AUC value, senescence scores were established for each cell. The function “AUCell_exploreThresholds” calculated the threshold that could deem the current gene-set active (Fig 11C). Then, cell clustering UMAP embeddings were colored according to each cell’s senescence score to indicate active cell clusters (Fig 11E). Corresponding violin plot revealed the highest scores in Cycling cells and the lowest in PT cells (Fig 11D). Moreover, senescence scores were markedly higher in the rejection group compared to the non-rejection group, clearly showcasing the significant role cellular senescence plays in influencing KTx rejection (Fig 11F).

## Discussion

Despite the evolution and sophisticated use of immunosuppressive medications, allograft rejection continues to significantly challenge of graft survival following renal transplantation. The exact mechanisms behind this phenomenon remain elusive, and the diagnosis of allograft rejection predominantly depends on invasive biopsy procedures, which remain the gold standard despite their invasiveness. Currently, shortage of organs necessitates the need to reassess donor eligibility criteria, with a particular focus on older donors. Notably, donor age emerges as a pivotal risk factor influencing the outcome of kidney transplants, where cellular senescence—traditionally characterized by a stable arrest in the cell cycle—is recognized as a fundamental marker of aging [5]. Research indicates that transplantation can initiate cellular senescence, with ischemia-reperfusion injury (IRI) notably inducing such changes, leading to senescent cell accumulation. This process is shown to compromise graft longevity, highlighting the intricate relationship between transplantation-induced stress and graft survival [46]. However, the intricate effects of cellular senescence on kidney allograft rejection are not well-understood. In this comprehensive study, we leveraged multiple public datasets from the GEO database along with various bioinformatic approaches to establish a direct correlation between cellular senescence and an increased rate of rejection, inversely affecting graft survival. Firstly, we identified SRGs linked to kidney rejection, and through clustering based on SRG expression profiles, discerned patient subclasses with unique molecular and clinical characteristics. Our analysis revealed two sets of four critical pSRGs instrumental for a peripheral blood diagnostic model and a graft survival prognostic model, respectively. Furthermore, the expression of these SRGs and their relationship with senescence and rejection were corroborated using single-cell dataset analysis, enriching our understanding of the complex dynamics at play in renal transplant rejection.

The concept of cellular senescence, first identified by Hayflick and Moorhead in the 1960s, describes the phenomenon where human diploid fibroblasts reach a limit in cell divisions, leading to growth arrest [47]. This process, pivotal in tissue remodeling, wound repair, and embryogenesis, becomes detrimental when prolonged, contributing to injury, cancer, and aging-related pathologies [48]. Recent research has extended to its implications in kidney transplantation, revealing that pre-transplant levels of senescent cells correlate with adverse outcomes such as interstitial fibrosis and chronic allograft nephropathy [49–51]. The presence of senescence markers, such as p21cip1 and p16ink4a, in pre-transplant biopsy specimens is linked to adverse outcomes [2,52]. Additionally, the genes CFHR1 and CFHR3, associated with the complement factor H family, have been identified as playing a role in inducing cellular senescence in the tubular cells of kidney allografts in patients with IgA nephropathy [53].

Allograft rejection significantly correlates with adverse outcomes, yet the role of senescence remains contentious. Clinically, debates persist regarding rejection risks in elderly recipients, highlighted by divergent findings in case series and reports [9]. Experimentally, the relationship between SASP and post-transplant immune responses is quite intricate but indispensable, with rejected grafts showing increased senescence markers, correlating with chronic nephropathy severity [54]. Evidence indicates that parenchymal changes in older allografts contribute to increased immunogenicity, leading to accelerated rejection [50,55]. Extracellular vesicles in antibody-mediated rejection can prompt senescence and the endothelial-to-mesenchymal transition in renal cells [56]. Also, senescence extends to the immunological level, causing "inflamm-aging" and altering immune responses in aged organ transplants [57]. Conversely, immunosenescence of CD4^+^ T cells and defective CD8 signaling pathways might enhance kidney allograft acceptance [58,59]. Therefore, understanding the nuanced interplay between cellular senescence and rejection is vital for optimizing post-transplant care and immunosuppressive strategies, particularly for elderly recipients. In this study, we conducted a differential analysis on RNA sequencing data from 282 renal transplant biopsy samples, including 76 with rejection and 206 without. GSEA analysis revealed significant enrichment in cellular senescence pathways. Intersection with WGCNA identified genes most associated with rejection, and subsequent GO and KEGG analyses of these hub genes confirmed their significant enrichment in aging and cellular senescence pathways, indicating a strong link between rejection and cellular aging. We further classified KTx samples into two clusters using consensus clustering methods, finding that cluster C2, more closely associated with aging, showed a higher rate of rejection and worse clinical outcomes. This suggests cellular senescence as a key factor in rejection and prognosis. Additionally, single-cell analysis with the AUCell package corroborated higher senescence scores in rejection samples, supporting our conclusions.

After demonstrating the macroscopic effects of senescence on rejection, we focused on SRGs associated with rejection. Differential and WGCNA analyses yielded 33 hub DE-SRGs. The results of functional enrichment analysis revealed significant associations of them with key biological pathways: NF-kappa B signaling, TNF signaling, and cytokine-cytokine receptor interactions. TNF, widely expressed across various tissues, triggers a classic inflammatory response upon binding with its receptors, including NF-κB activation and the expression of pro-inflammatory cytokines [60]. In the kidney, TNFα plays a role in the radiation-induced senescence of kidney epithelial cells [61]. Moreover, The NF-kB pathway plays a crucial role in regulating cellular senescence and SASP observed in various degenerative diseases, and inhibiting this pathway can effectively delay the onset of DNA damage-induced senescence and aging in mice [62,63].

Subsequently, using SVM and RF machine learning methods, we identified 13 pSRGs consistently upregulated in rejection samples across the discovery and two external datasets. As kidney transplant rejection typically manifests through serum creatinine alterations after significant damage, establishing non-invasive, effective predictive methods has been a goal for researchers. Previous studies highlight that molecular changes in peripheral circulating cells accompany kidney tissue alterations in transplant injury processes. Validating pSRGs in peripheral blood of KTx samples revealed significant downregulation of IL2RB, IL15, and TNFSF13B in rejection samples, with upregulation of C1QA. With RF model ultimately being chosen for its superior evaluation performance, this 4-gene assay demonstrated robust diagnostic accuracy in distinguishing between cases with and without rejection, achieving AUC values of 97.5% and 74.9%. Furthermore, the constructed nomogram showcased excellent clinical predictive performance (AUC 89.7%), underscoring its potential utility in the clinical setting for assessing the risk of rejection in transplant patients.

To further investigate whether SRGs impact the prognosis of transplanted kidneys through rejection, we initially utilized the UniCox method to exclude SRG irrelevant to prognosis. Subsequently, we employed the LASSO technique to construct a 4-SRG prognostic model (comprising ACKR1, CCL4, STAT1, MAPK1) for evaluating long-term outcomes in kidney transplants. The time-dependent ROC analysis showcased the predictive capability of the 4-SRG signature, highlighting its effectiveness in forecasting outcomes. As for the four SRGs included in our model, ACKR1, also known as Atypical Chemokine Receptor 1 or DARC, plays a crucial role in chemokine regulation, modulating angiogenic activity in endothelial cells and potentially reducing malignancy risk in high-risk lesions [64,65]. In KTx, ACKR1 expression increases during acute rejection and crescentic glomerulonephritis [66]. CCL4, vital for leukocyte recruitment and activation, shows elevated levels with aging and is universally linked to organ allograft rejection [67,68]. STAT1, essential in the JAK-STAT pathway, induces senescence in human glomerular mesangial cells [69–71]. The ERK/MAPK pathway, involved in various cellular processes including senescence, has been implicated in chronic antibody-mediated rejection (cABMR) through single-cell transcriptome analysis [72]. Furthermore, Our analysis of single-cell sequencing data from transplanted kidneys shows significant differential expression of ACKR1 and CCL4 between grafts with stable function and those undergoing rejection. This suggests their potential value and warrants further investigation into their roles in transplant outcomes.

Certainly, like any research, our study has its limitations. Firstly, our findings based on bioinformatics analyses need validation through experimental studies and clinical sample collection. Secondly, kidney transplant rejection has various subclasses, such as Acute vs. Chronic and AntiBody-Mediated vs. T-Cell-Mediated Rejection. Our study only differentiates based on the occurrence of rejection without specifying the type. Thirdly, a broader dataset with comprehensive follow-up data on renal transplants would enhance our prognostic model, potentially integrating clinical parameters for more accurate predictions. Additionally, the datasets used in this study were primarily from European and North American cohorts, which may limit the generalizability of our findings to other populations, such as Asian cohorts. Future studies should aim to validate these biomarkers across more diverse populations to ensure their robustness. Lastly, our functional enrichment and immune infiltration analysis results could pave the way for further exploration into how SRGs regulate senescence and impact kidney transplant rejection at the cellular and molecular levels. This could offer valuable insights for developing targeted interventions to improve transplant outcomes.

In conclusion, we comprehensively explored the role of senescence in kidney transplant rejection, underscoring the significant function of key SRGs in diagnosing rejection and forecasting adverse prognoses. Our findings include: 1) a predictive medical approach using 13 pSRGs as potential biomarkers for KTx rejection, among which IL2RB, IL15, TNFSF13B, and C1QA were used to develop a robust, non-invasive signature for early detection of acute rejection (AR). Overexpression of ACKR1, CCL4, and STAT1 post-KTx indicates a higher risk of graft loss, while MAPK1 suggests a protective effect. 2) a focus on targeted prevention and secondary prevention strategies based on the expression of pSRGs, reflecting the prognostic and immunological characteristics of KTx patients, aiming to intercept the transition from pre-clinical to clinical phases of rejection; and 3) the suggestion of personalized treatment strategies that consider early identification of cellular senescence for patient clustering and risk assignment, enhancing the efficacy of immunosuppressive therapy and mitigating graft dysfunction, especially in high-risk patients.

## Supporting information

S1 Fig WGCNA analysis of GSE21374. **A.** Cluster Dendrogram: Hierarchical clustering of genes, with branches representing gene modules, color-coded below the dendrogram. These modules reflect co-expressed genes potentially involved in transplant rejection. **B.** Scale Independence and Mean Connectivity: Left: Scale-free topology model fit (R^2) versus soft-thresholding power, ensuring network adherence to a scale-free topology. Right: Mean connectivity as a function of soft-thresholding power, used to determine the optimal network parameters. **C.** Network Heatmap: Topological overlap matrix (TOM) heatmap showing the strength of connections between genes, with dendrograms and module colors corresponding to (**A**).
(TIF)

S2 Fig Reverse cumulative distribution and boxplot of residual for model performance comparison. (TIF)

S3 Fig Quality control and batch effect removal of scRNA analysis. (TIF)

S1 Table Senescence/Aging-related pathways for GSVA analysis. (CSV)

S1 File (ZIP)
