# Supplementary figures and images for: Deciphering the impact of senescence in kidney transplant rejection: An integrative machine learning and multi-omics analysis via bulk and single-cell RNA sequencing
Source: PLoS One. 2024 Nov 27;19(11):e0312272. doi: 10.1371/journal.pone.0312272 (PMC11602102; doi:10.1371/journal.pone.0312272)

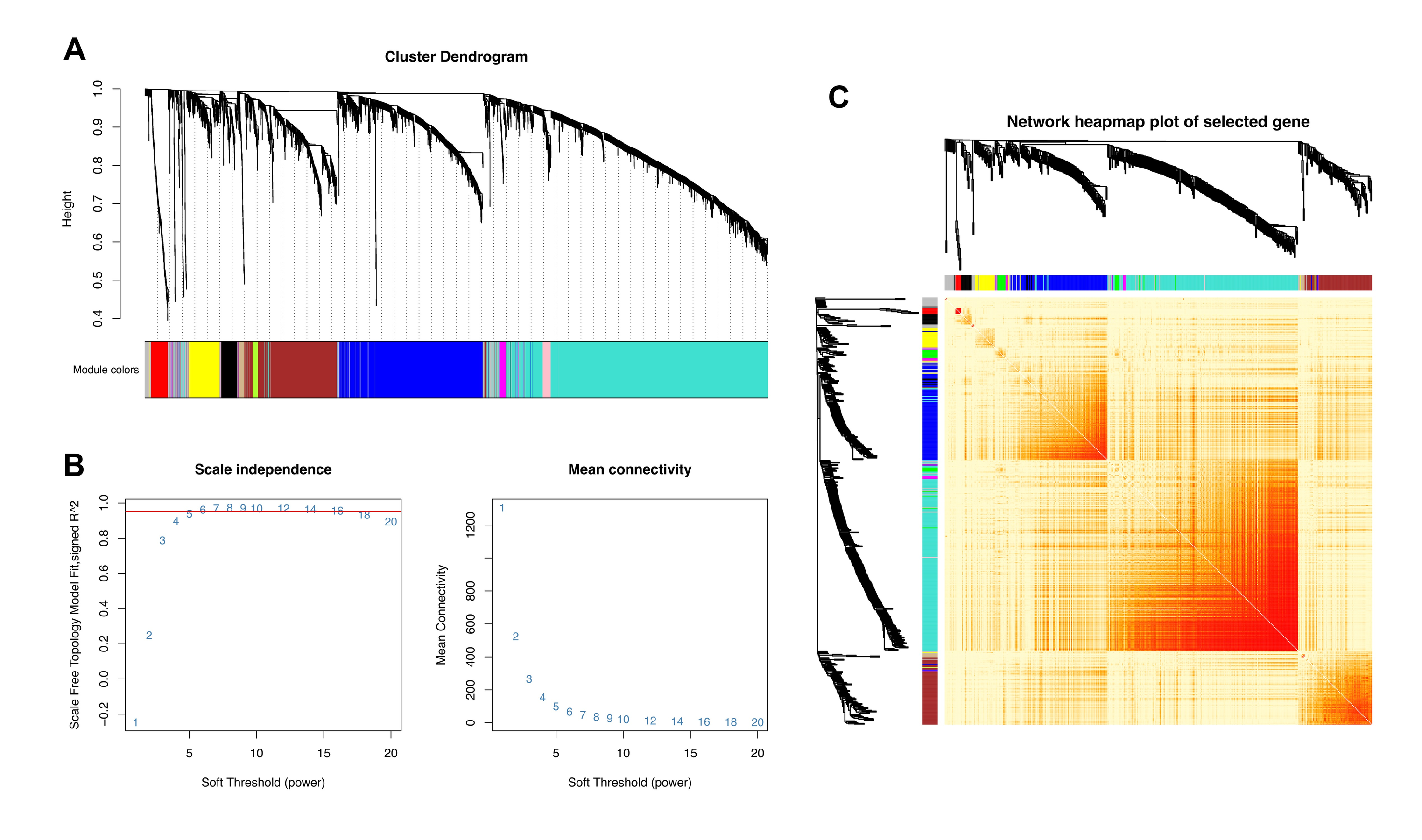

Supplement: S1 Fig — A. Cluster Dendrogram: Hierarchical clustering of genes, with branches representing gene modules, color-coded below the dendrogram. These modules reflect co-expressed genes potentially involved in transplant rejection. B. Scale Independence and Mean Connectivity: Left: Scale-free topology model fit (R^2) versus soft-thresholding power, ensuring network adherence to a scale-free topology. Right: Mean connectivity as a function of soft-thresholding power, used to determine the optimal network parameters. C. Network Heatmap: Topological overlap matrix (TOM) heatmap showing the strength of connections between genes, with dendrograms and module colors corresponding to (A). (TIF) [file pone.0312272.s001.tif]

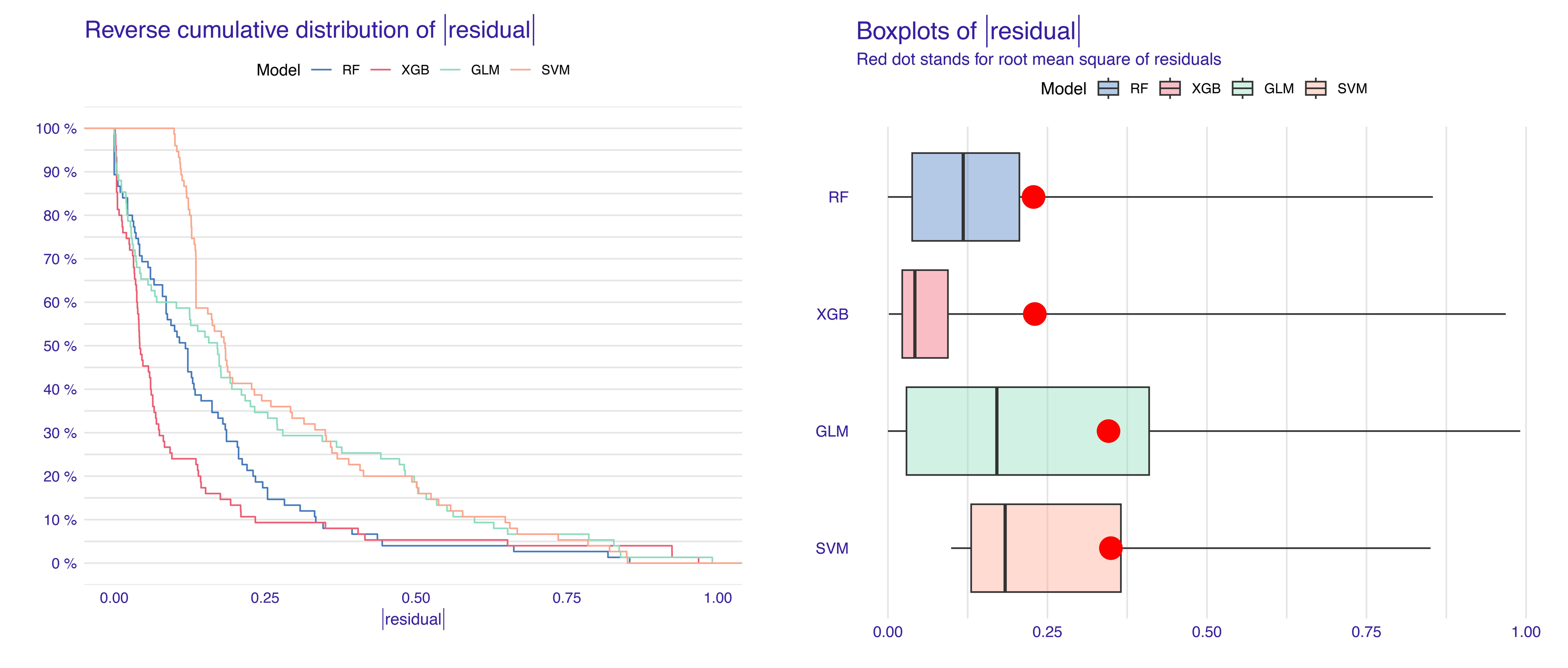

Supplement: S2 Fig — (TIF) [file pone.0312272.s002.tif]

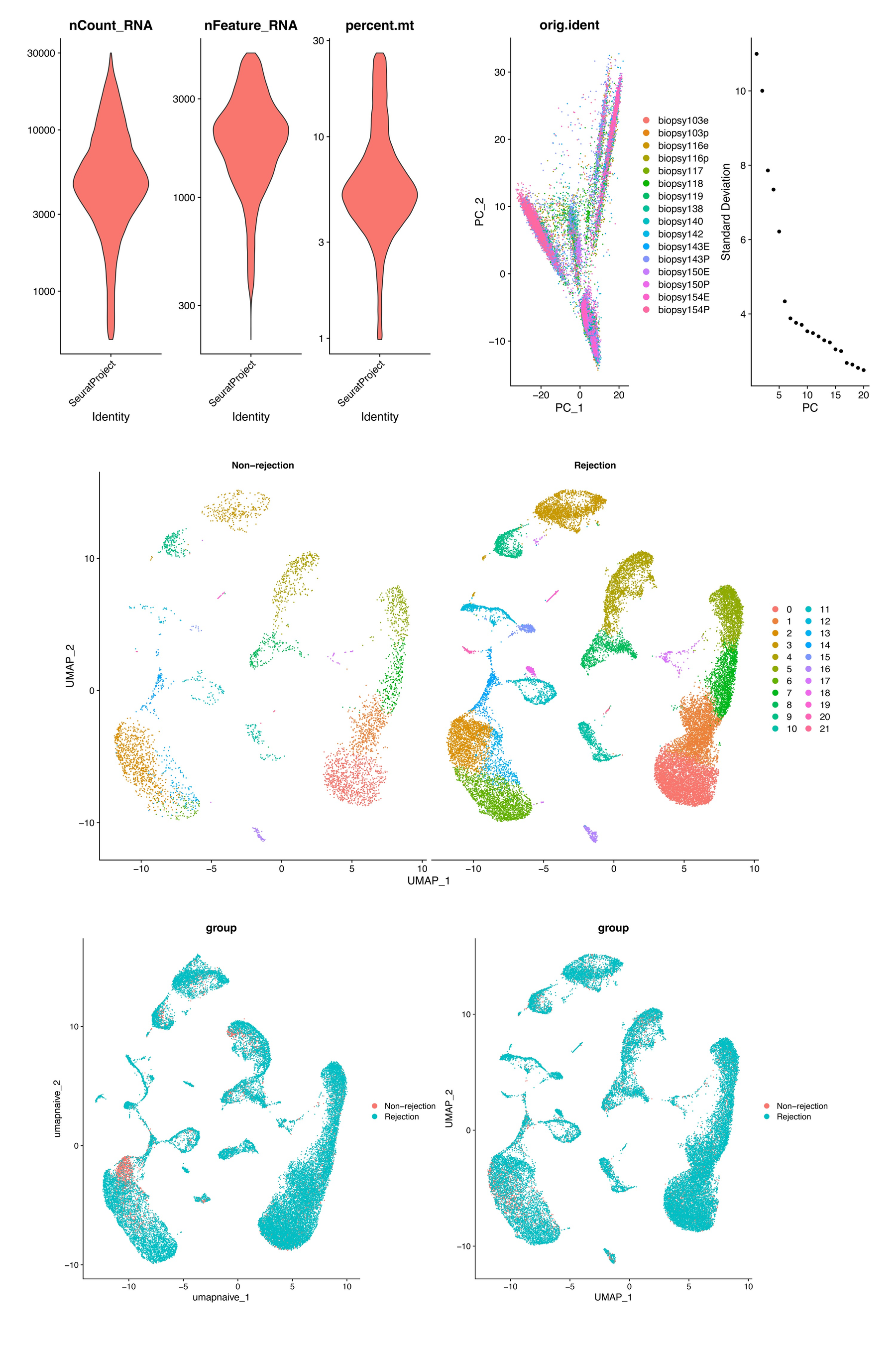

Supplement: S3 Fig — (TIF) [file pone.0312272.s003.tif]
